# Yield and predictors of conversion on serial amyloid nuclear SPECT/CT in at-risk populations for transthyretin cardiac amyloidosis

**DOI:** 10.1093/ehjimp/qyag089

**Published:** 2026-05-16

**Authors:** Saberio Lo Presti, Amr Darwish, Zoran Popovic, Mohamed Awad, Bryan Abadie, Tom Wang, Wael Jaber, Mazen Hanna

**Affiliations:** Section of Advanced Cardiac Imaging, Department of Cardiovascular Medicine, Heart, Vascular & Thoracic Institute, Cleveland Clinic, Cleveland, OH, USA; Department of Internal Medicine, Cleveland Clinic Fairview Hospital, Cleveland, OH, USA; Section of Advanced Cardiac Imaging, Department of Cardiovascular Medicine, Heart, Vascular & Thoracic Institute, Cleveland Clinic, Cleveland, OH, USA; Department of Anesthesiology and Critical Care, Ain Shams University, Cairo, Egypt; Section of Advanced Cardiac Imaging, Department of Cardiovascular Medicine, Heart, Vascular & Thoracic Institute, Cleveland Clinic, Cleveland, OH, USA; Section of Advanced Cardiac Imaging, Department of Cardiovascular Medicine, Heart, Vascular & Thoracic Institute, Cleveland Clinic, Cleveland, OH, USA; Section of Advanced Cardiac Imaging, Department of Cardiovascular Medicine, Heart, Vascular & Thoracic Institute, Cleveland Clinic, Cleveland, OH, USA; Section of Heart Failure and Cardiac Transplant, Department of Cardiovascular Medicine, Heart, Vascular, & Thoracic Institute, Cleveland Clinic, Cleveland, OH, USA

**Keywords:** transthyretin amyloidosis, cardiac amyloidosis, amyloid scintigraphy, serial imaging, technetium-labelled tracers

## Abstract

**Aims:**

Transthyretin cardiac amyloidosis (ATTR-CA) is an increasingly recognized cause of heart failure, yet data guiding surveillance strategies in high-risk individuals remain limited. This study evaluated the incidence, imaging patterns, and clinical predictors of SPECT/CT conversion among patients undergoing serial Tc-99m PYP/HMDP imaging.

**Methods and results:**

We conducted a retrospective cohort study of 106 patients who underwent repeat Tc-99m PYP or HMDP cardiac SPECT/CT between 2018 and 2025. Patients with a positive initial scan or repeat imaging within less than 12 months were excluded. Conversion was defined as a new Perugini grade ≥1 with any myocardial radiotracer uptake on follow-up. Clinical characteristics, biomarkers, genetic testing, and extracardiac biopsy findings were analysed. Univariable and multivariable Cox regression identified predictors of conversion. Over a mean follow-up of 3.2 years, 14 patients (13.2%) showed imaging conversion. Echocardiographic parameters and absolute biomarker levels were comparable between groups; however, a ≥30% rise in NT-proBNP was more frequent among converters (71.4% vs. 38.1%, *P* = 0.018). On univariable analysis, hypertension was associated with a lower hazard of conversion (HR 0.27; *P* = 0.023), while co-occurrence of carpal tunnel syndrome and spinal stenosis was associated with a significantly higher hazard (HR 3.90; *P* = 0.024). In multivariable analysis adjusted for age and sex, this musculoskeletal phenotype remained independently associated with conversion (adjusted HR 5.95; *P* = 0.019).

**Conclusion:**

The combined presence of carpal tunnel syndrome and spinal stenosis identifies a high-risk phenotype for future imaging conversion. These exploratory findings support serial amyloid SPECT/CT for surveillance and highlight the value of musculoskeletal comorbidities in refining risk stratification for ATTR-CA.

## Graphical Abstract

**Graphical Abstract:**
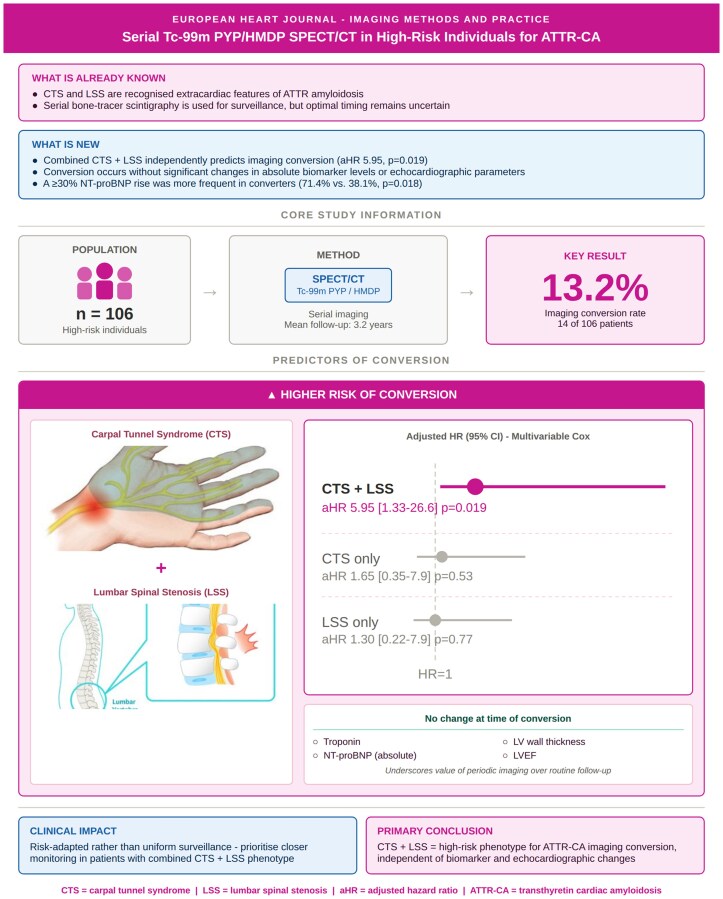
Amyloid SPECT/CT Conversion in patients at risk for ATTR-CA. Among 106 at-risk individuals with an initially negative scan, 13% developed imaging conversion over a mean follow-up of 3.2 ± 1.3 years. Conversion occurred predominantly beyond the second year of surveillance. The co-occurrence of carpal tunnel syndrome and lumbar spinal stenosis identified a musculoskeletal phenotype associated with increased hazard of conversion, whereas hypertension was associated with lower risk. These findings support individualized surveillance strategies in patients with extracardiac manifestations of transthyretin amyloidosis.

## Introduction

Cardiac amyloidosis (CA) is a restrictive cardiomyopathy caused by the extracellular deposition of amyloid fibrils within the myocardium.^1,2^ Transthyretin amyloidosis (ATTR) is a leading cause of CA, particularly among older adults, and occurs in two forms: hereditary (hATTR) due to pathogenic *TTR* variants, and wild-type (wtATTR), which develops sporadically.^1^ Although endomyocardial biopsy remains the diagnostic gold standard, its invasive nature and associated risks have made it less favoured in contemporary clinical practice^1–3^

The diagnostic approach to ATTR-cardiac amyloidosis (ATTR-CA) has been transformed by bone-avid radiotracer scintigraphy, most commonly using technetium-99m pyrophosphate (Tc-99m PYP) or technetium-99m hydroxymethylene diphosphonate (Tc-99m HMDP) in the USA.^4^ These tracers bind to calcium-phosphate complexes associated with transthyretin fibrils, enabling non-biopsy confirmation of ATTR-CA with high specificity when monoclonal protein testing is negative.^4^ The Gillmore non-biopsy criteria, established in *Circulation* in 2016, validated the use of grade ≥ 2 myocardial uptake on planar scintigraphy—provided light-chain amyloidosis is excluded—as sufficient for diagnosing ATTR-CA.^2^ Subsequent integration of single photon emission computed tomography combined with low dose computed tomography (SPECT/CT) imaging has further enhanced diagnostic specificity by confirming true myocardial uptake and reducing misclassification related to blood-pool activity or rib artefact, an effect strengthened by CT-based attenuation correction and improved anatomic delineation.^4,5^ As a result, PYP/HMDP SPECT/CT has become central not only to diagnosis but also to screening individuals at elevated risk for early detection of ATTR-CA.^4^

Certain populations are recognized as being at risk for developing ATTR-CA, making serial Tc-99m PYP/HMDP testing a frequent part of surveillance. The first group comprises patients with extracardiac biopsy–proven amyloidosis, often identified during carpal tunnel release or spinal decompression surgery through tenosynovial or ligamentum flavum specimens^6–9^. The second group includes individuals with transthyretin amyloid detected in left atrial appendage (LAA) tissue obtained incidentally during cardiac surgery—an increasingly recognized early marker of systemic ATTR amyloid deposition that may precede ventricular involvement.^10,11^ The third group consists of asymptomatic carriers of cardiac-dominant *TTR* variants (ATTRv).^12^ An additional subset of patients also undergoes repeat imaging based on high clinical suspicion derived from clinical, echocardiographic, and epidemiologic features. Expert consensus recommends annual clinical evaluation and periodic assessment for cardiac involvement with Tc-99m PYP/HMDP imaging every 3–5 years in individuals with a genetic predisposition.^12^ However, these interval-based recommendations have been broadly extrapolated to other at-risk groups despite limited evidence to support optimal timing or the clinical utility of repeat radionuclide imaging.

The overall aim of this study is to evaluate the role of serial PYP/HMDP SPECT/CT in detecting cardiac involvement among distinct high-risk populations. Specifically, the study seeks to characterize patient demographics and clinical profiles, quantify rates and predictors of conversion to ATTR-CA, and establish evidence-based recommendations for safe and effective intervals for repeat Tc-99m PYP/HMDP screening.

## Methods

### Study design and population

This was a retrospective, observational cohort study conducted under an Institutional Review Board (IRB)–approved protocol at the Cleveland Clinic. Because of its retrospective design and de-identified data extraction, the requirement for informed consent was waived.

We included adult patients (≥18 years) who underwent at least two diagnostic PYP or HMDP SPECT/CT between January 2018 and March 2025 at high risk of developing cardiac amyloidosis. Patients were classified as high-risk for transthyretin cardiac amyloidosis (ATTR-CA) if they met at least one of the following criteria: (i) histologically confirmed amyloid deposits in extracardiac tissue such as carpal tunnel tenosynovium or ligamentum flavum, (ii) left atrial appendage (LAA) amyloid deposition identified during cardiac surgery; (iii) the presence of a documented transthyretin gene variant (e.g. p.Val142Ile, p.T80A), (iv) high suspicion based on clinical, echocardiographic, and epidemiological findings. Patients were excluded if they had incomplete imaging data, non-diagnostic studies, positive baseline scans, or if the follow-up scan occurred within a year.

All patients underwent planar and SPECT/CT following intravenous injection of Tc-99m PYP (15–25 mCi) or Tc-99m HMDP (15–20 mCi) using standardized acquisition and interpretation parameters.^2,4,5,13^ Images were obtained 3 h post-injection using dual-head SPECT cameras with additional low-dose CT acquisition for attenuation correction of artefacts and enhanced anatomical delineation. Visual myocardial uptake was graded according to the Perugini scale (0 = none; 1 = less than bone; 2 = equal to bone; 3 = greater than bone).^2,4,5,13^ In the absence of a monoclonal protein, scans demonstrating Perugini grade ≥1 myocardial uptake on planar images with corresponding uptake on SPECT/CT images were considered diagnostic for ATTR-CA.^2,4,13^ Imaging conversion was defined as a change from a previously negative scan to a new Perugini grade ≥1 with any myocardial tracer uptake on SPECT/CT at follow-up imaging, irrespective of the number of intervening scans or the time interval between studies.^13,14^ It is important to note that scintigraphy conversion may represent early or subclinical amyloid deposition rather than overt, clinically manifest ATTR-CA. However, these findings may still be meaningful, as they can identify patients at a stage where treatment may help prevent disease progression. Two experienced nuclear cardiologists (SLP, WJ) reviewed all scans independently while blinded to clinical data, and discrepancies were resolved by consensus.

Demographic and clinical variables were obtained from the electronic health record and registry. Baseline covariates included age, sex, hypertension, diabetes mellitus, chronic kidney disease (CKD ≤ stage III), and indication for initial scan [e.g. cardiomyopathy, carpal tunnel syndrome (CTS), lumbar spinal stenosis (LSS), extracardiac tissue amyloid proven biopsy]. Laboratory variables, such as high-sensitivity troponin, NT-proBNP, and estimated glomerular filtration rate (eGFR), were extracted within one month of each scan (baseline and follow-up). A ≥30% increase in NT-proBNP was selected *a priori* to represent a clinically meaningful change that exceeds expected biological and analytical variability and has been commonly used in heart failure literature to define significant biomarker worsening over time.^15^ Echocardiographic parameters (LV septal and posterior wall thickness, ejection fraction, apical sparing) and ECG findings (low-voltage QRS) were recorded when available. For those with tissue biopsies, histopathologic sites (carpal tunnel, ligamentum flavum, LAA) and amyloid typing were documented. Genetic testing results for TTR mutations were recorded when available. Exposure to disease-modifying agents such as tafamidis, diflunisal, or vutrisiran was recorded at follow-up.

### Outcomes

The primary outcome was conversion on serial SPECT/CT, defined as the change from a prior negative scan to a subsequent grade ≥1 scan on planar images and any degree of tracer deposit in the myocardium on SPECT/CT images. Secondary outcomes included the time interval between the first and subsequent scans, identification of clinical and biomarker predictors associated with conversion.

### Statistical analysis

Demographic and clinical characteristics were summarized as frequencies (percentages) for categorical variables and as mean ± standard deviation (SD) or median [interquartile range (IQR)] for continuous variables, as appropriate. Between-group comparisons were performed using the chi-square or Fisher’s exact test for categorical variables and the Wilcoxon rank-sum test for continuous variables.

Time-to-event analyses were performed using Cox proportional hazards regression to identify predictors of scintigraphic scan conversion. Univariable Cox models evaluated age, sex, hypertension, diabetes mellitus, chronic kidney disease, biomarker levels (troponin and NT-proBNP), aortic stenosis, biopsy status, CTS, LSS, and a binary variable representing the co-occurrence of CTS and LSS.

A multivariable Cox model was constructed, including age and sex (pre-specified), hypertension (selected based on univariable association), and a four-level musculoskeletal category (neither condition , CTS only, LSS only, and both conditions) to estimate independent and joint effects while minimizing collinearity. Hazard ratios (HRs) with 95% confidence intervals (CIs) were reported. All the analyses were performed on Stata version 17.0 (StataCorp LLC, College station, TX, USA). A *P* value of <0.05 was considered statistically significant.^16^

## Results

### Study population

**Figure 1:**
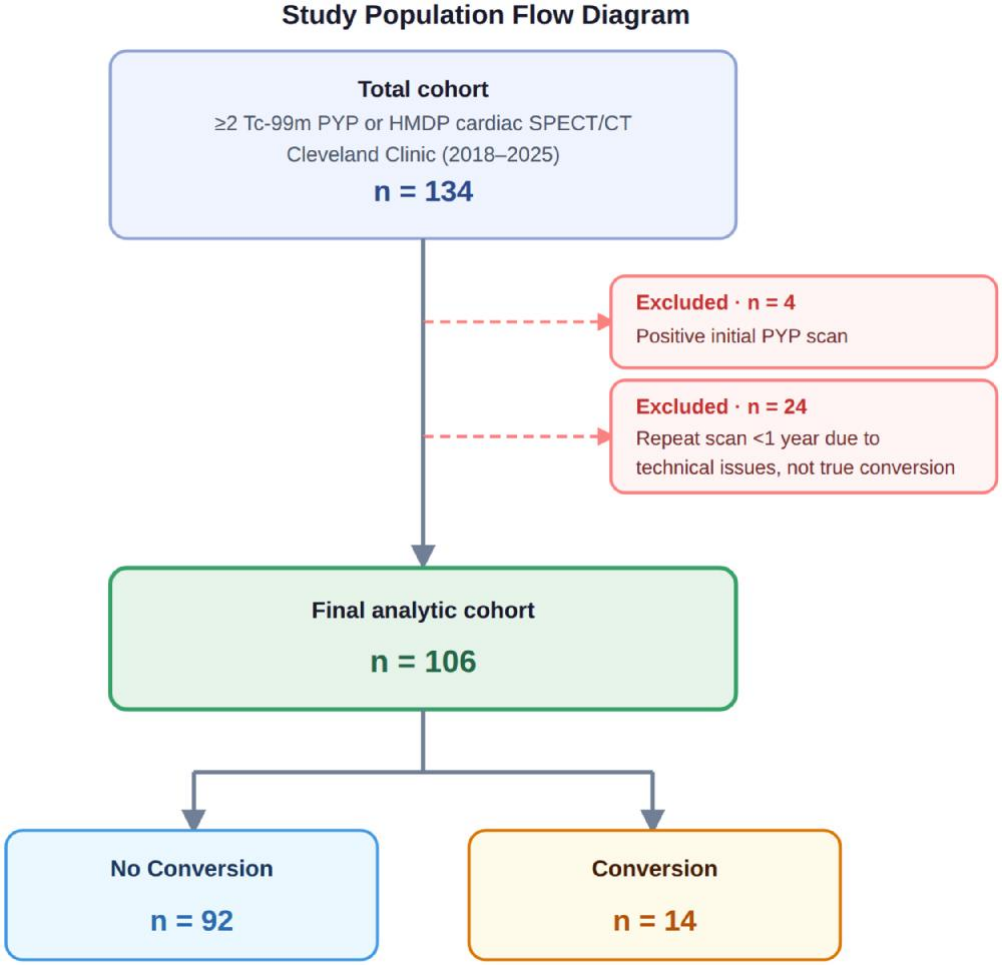
Study flow diagram. Among 134 patients undergoing ≥ Tc-99m PYP or HMDP Cardiac SPECT/CT at the Cleveland Clinic (2018–2025), 4 were excluded for a positive initial scan and 24 were excluded for repeat imaging within 12 months, for concerns of technical variability. The final cohort included 106 patients: 92 showed no conversion and 14 showed conversion. *Conversion was defined as new Perugini grade ≥ 1 myocardial uptake on planar imaging and any uptake on SPECT/CT.*

Between January 2018 and March 2025, a total of 134 patients underwent serial Tc-99m PYP or HMDP cardiac SPECT/CT for evaluation of suspected transthyretin cardiac amyloidosis. Four patients were excluded because the baseline (first) scan was already positive, precluding assessment of conversion. An additional 24 patients were excluded because the interval between baseline and follow-up scans was less than 365 days. Repeating imaging within such a short timeframe was felt to be more reflective of technical variability or image-quality concerns rather than true biological disease progression, and these patients were therefore excluded *a priori* from the conversion analysis (*Figure 1*).

Among the 106 included high-risk patients, 14 (13.2%) demonstrated imaging conversion on follow-up. The mean interval between baseline and follow-up scans in the overall cohort was 1087 ± 525 days, 3.0 ± 1.4 years. Patients who converted had a numerically longer interval between scans compared with non-converters (1171 ± 500 vs. 1075 ± 530 days, 3.2 ± 1.3 years vs. 2.9 ± 1.4 years). Only 14% of conversions occurred during the second year, whereas the remaining occurred during the 3–5 years interval (see Supplementary data online, *Table S1*).

### Baseline characteristics and indications for scanning

**Figure 2:**
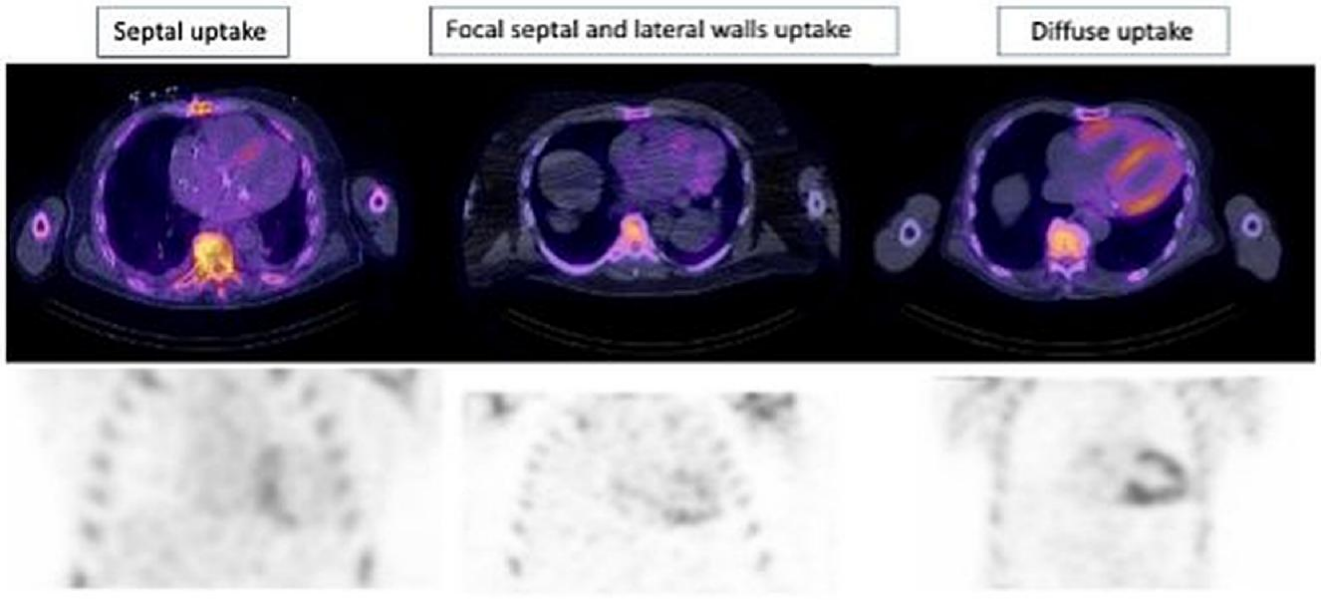
Patterns of uptake with positive conversion. Upper row: (Left panel) Focal uptake in the septum; (Middle Panel) focal uptake in the septum and lateral wall; (Right Panel) diffuse uptake, extending to the right ventricular free wall. Lower row: Corresponding planar data, demonstrating radiotracer uptake equal or greater than rib uptake (grade ≥1), with similar distribution demonstrated on SPECT/CT images.

Baseline clinical and imaging characteristics are summarized in *Table 1*. The mean age was 73.9 ± 11.9 years, and 31.1% of patients were female. Patients who converted were older on average than non-converters (77.9 ± 10.0 vs. 73.2 ± 12.1 years), though this difference was not statistically significant (*P* = 0.288). Among the 14 patients who demonstrated imaging conversion, patterns of myocardial uptake varied on SPECT/CT. Early, focal septal uptake was observed in 4 patients (29%), intermediate uptake involving the septum and lateral wall in 4 patients (29%), and diffuse myocardial uptake in 6 patients (43%) (*Figure 2*).

**Table 1:** Baseline clinical and imaging characteristics

Variable	Total (*n* = 106)	No conversion (*n* = 92)	Conversion (*n* = 14)	*P* value
Clinical characteristics				
Age at last scan, years (mean, SD)	73.85 ± 11.86	73.23 ± 12.05	77.87 ± 10.02	0.288
Female sex, *n* (%)	33 (31.1)	29 (31.5)	4 (28.6)	1.00
Hypertension, *n* (%)	77 (72.6)	70 (76.1)	7 (50.0)	**0.041**
Diabetes mellitus, *n* (%)	27 (25.5)	24 (26.1)	3 (21.4)	1.00
CKD (≤ stage III), *n* (%)	51 (48.1)	43 (46.7)	8 (57.1)	0.468
Electrocardiographic variables				
Low-voltage ECG, *n* (%)	7 (6.6)	6 (6.5)	1 (7.2)	1.00
Echocardiographic variables				
Apical sparing on TTE, *n* (%)	9 (8.5)	8 (8.7)	1 (7.2)	1.00
Septal wall thickness, cm (mean, SD)	1.20 ± 0.36	1.20 ± 0.34	1.39 ± 0.44	0.242
Posterior wall thickness, cm (mean, SD)	1.09 ± 0.27	1.09 ± 0.27	1.14 ± 0.24	0.688
LVEF, % (mean, SD)	56.7 ± 11.2	56.7 ± 11.3	56.8 ± 11.3	0.665
Imaging interval				
Interval between scans, days (mean, SD)	1087 ± 525	1075 ± 530	1171 ± 500	0.41
Laboratory variables—baseline (first scan)				
Troponin, ng/L (median, IQR)	32 [22–66]	32 [20–68]	48 [30–66]	0.787
NT-proBNP, pg/mL (mean, IQR)	524.5 [105–2215]	524.5 [99–2283]	698.5 [155–1739]	0.992
eGFR, mL/min (median, IQR)	62.5 [44–84]	57 [44–84]	74 [46–86]	0.413
Laboratory variables—follow-up (second scan)				
Troponin, ng/L (median, IQR)	26.5 [19–50.5]	26 [17–47]	32 [23–56]	0.265
NT-proBNP, pg/mL (median, IQR)	467 [123–3844]	448 [132–2507]	1497.5 [267–7065]	0.17
eGFR, mL/min (median, IQR)	59 [42–82]	59.5 [40–82.5]	56 [46–76]	0.78
≥ 30% increase in NTproBNP, *n* (%)	45 (42.5)	35 (38.1)	10 (71.4)	**0.018**

Statistically significant difference was found between comparison groups with p value less than 0.05.

Hypertension was significantly less prevalent among converters (50% vs. 76.1%, *P* = 0.041). Prevalence of diabetes mellitus, chronic kidney disease (stage III or higher), and low-voltage ECG findings were similar between groups. Echocardiographic parameters, including left ventricular ejection fraction, septal and posterior wall thickness, and the presence of apical sparing, did not differ significantly between converters and non-converters. Baseline cardiac biomarkers, including high-sensitivity troponin, NT-proBNP, and eGFR, were comparable between groups at both the first and second scans (all *P* > 0.05). A ≥30% increase in NT-proBNP occurred more frequently among converters than non-converters (71.4% vs. 38.1%, *P* = 0.018) (*Table 1*).

The primary clinical indications for obtaining a Tc-99m PYP/HDMP scans, as documented in the medical record by the referring physicians, are summarized in *Table 2*. Baseline clinical characteristics were otherwise similar between groups, with no significant differences in most individual indications. However, the combined presence of CTS and LSS was significantly more frequent in the conversion group (28.6% vs. 4.5%, *P* = 0.01), whereas isolated musculoskeletal manifestations were not associated with conversion.

**Table 2:** Clinical indications for serial Tc-99m PYP/HMDP scintigraphy

Indication	Total (*n* = 106)	No Conversion (*n* = 92)	Conversion (*n* = 14)	*P* value
Heart failure/cardiomyopathy	24 (22.6)	20 (21.7)	4 (28.6)	0.516
LV hypertrophy (any)	10 (9.4)	10 (10.9)	0 (0)	0.352
Aortic stenosis	14 (13.2)	12 (13.0)	2 (14.3)	1.00
Carpal tunnel syndrome	29 (25.5)	25 (27.2)	4 (28.6)	1.00
Lumbar spinal stenosis	11 (10.4)	9 (9.8)	2 (14.3)	0.637
Carpel tunnel + lumbar spinal stenosis	8 (7.5)	4 (4.45)	4 (28.6)	**0.01**
Peripheral neuropathy	12 (11.3)	9 (9.8)	3 (21.4)	0.195
Abnormal TTE (strain)	8 (7.6)	7 (7.6)	1 (7.1)	1.00
Abnormal ECG	1 (1.0)	1 (1.0)	0 (0)	1.00
Family history of amyloidosis	6 (5.7)	6 (6.5)	0 (0)	1.00
Tissue biopsy positive	47 (44.3)	39 (42.3)	8 (57.1)	0.44

Statistically significant difference was found between comparison groups with p value less than 0.05.

Among the 47 patients who underwent tissue biopsy (44.3%), tenosynovial tissue was the most common biopsy site (68.1%), followed by ligamentum flavum (17.0%), left atrial appendage (6.4%), and other sites (8.5%) (see Supplementary data online, *Table S2*). Among patients who developed conversion (*n* = 14), 4 (28.6%) had a pathogenic TTR mutation, including 2 (14.3%) with Val142Ile and 2 (14.3%) with other variants, whereas the majority (71.4%) had wild-type disease (see Supplementary data online, *Table S3*). Conversion rates varied across the pre-specified at-risk subgroups (see Supplementary data online, *Table S4*). Among patients with extracardiac biopsy-proven amyloid, 8 of 47 (17.0%) demonstrated imaging conversion. Conversion was most frequent among TTR mutation carriers, occurring in 4 of 19 (21.1%) patients. In contrast, among patients referred on the basis of high clinical suspicion alone, conversion was observed in only 2 of 42 (4.8%). As subgroups were not mutually exclusive and the number of events per group was small, formal between-group comparisons were not performed; these values are presented descriptively to contextualize heterogeneity in baseline risk across the cohort. Regarding therapy, 9 patients (64.3%) ultimately received tafamidis, 1 (7.1%) diflunisal, and 3 (21.4%) vutisiran after conversion. Only 1 patient (7.1%) did not receive disease-modifying after conversion and unfortunately, he died shortly after establishing diagnosis (see Supplementary data online, *Table S3*).

**Figure 3:**
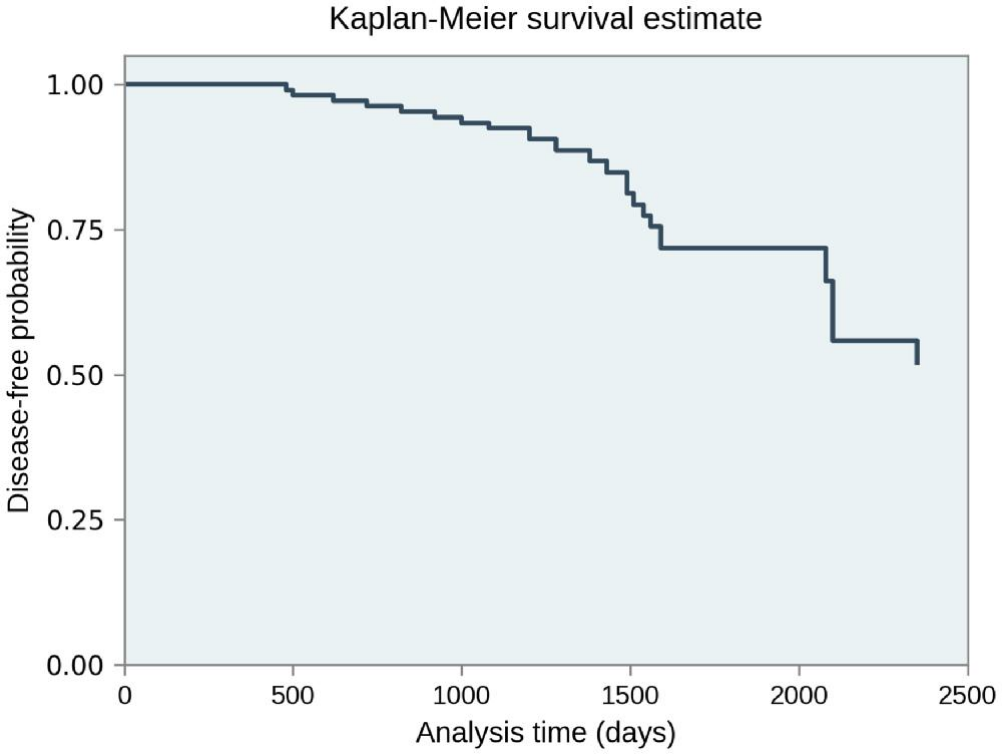
Kaplan–Meier estimate of conversion-free survival. Kaplan–Meier analysis demonstrating progressive decline in conversion-free survival over time, with the majority of events occurring beyond the first year of follow-up. The y-axis reflects the probability of remaining conversion-free, and the x-axis denotes analysis time (days).

Kaplan–Meier analysis, including all the screened patients demonstrated a gradual decline in conversion-free survival over follow-up. The survival curve progressively declined past the first year, with most events occurring between approximately 2 and 4 years (*Figure 3*).

### Predictors of SPECT/CT conversion

**Figure 4:**
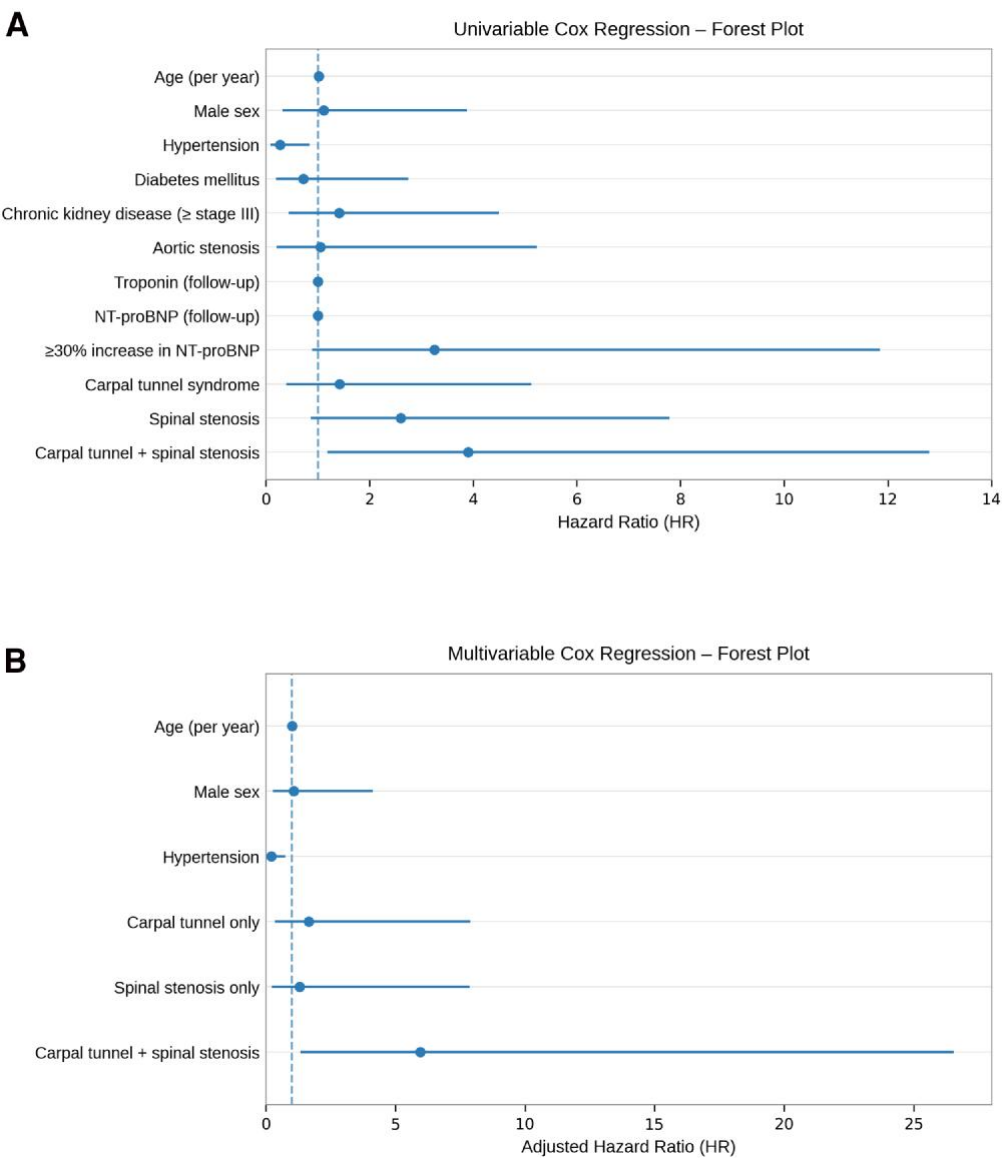
Predictors of imaging conversion. Panel A shows univariable Cox regression analyses of clinical characteristics and biomarker measures associated with conversion. Panel B shows the multivariable Cox regression model. Hazards ratios (HRs) with 95% CIs are displayed on a linear scale; the dashed line denotes HR = 1.

In univariable Cox regression, hypertension was associated with a significantly lower hazard of scan conversion (HR 0.27, 95% CI 0.09–0.84; *P* = 0.023). The co-occurrence of CTS and LSS was associated with a significantly higher hazard of conversion (HR 3.90, 95% CI 1.19–12.80; *P* = 0.024). LSS showed a nonsignificant trend toward increased hazard (HR 2.60, 95% CI 0.87–7.79; *P* = 0.088), and a ≥30% increase in NT-proBNP demonstrated a borderline association (HR 3.25, 95% CI 0.89–11.85; *P* = 0.074). Age, sex, diabetes mellitus, chronic kidney disease, aortic stenosis, and biomarker levels (troponin, NT-proBNP) were not significantly associated with conversion in univariable analyses. (*Table 3*; *Figure 4*, Panel A).

**Table 3:** Univariable cox regression for imaging conversion

Variable	Hazard ratio (HR)	95% CI	*P* value
Age (per year)	1.02	0.96–1.08	0.523
Male sex	1.12	0.32–3.88	0.859
Hypertension	0.27	0.09–0.84	**0.023**
Diabetes mellitus	0.72	0.19–2.75	0.636
Chronic kidney disease (≤ stage III)	1.41	0.44–4.50	0.566
Aortic stenosis	1.05	0.21–5.23	0.952
Troponin (follow-up)	1.00	0.98–1.02	0.823
NT-proBNP (follow-up)	1.00	0.999–1.00	0.472
≥30% increase in NT-proBNP	3.25	0.89–11.85	0.074
Carpal tunnel syndrome	1.42	0.39–5.12	0.588
Lumbar spinal stenosis	2.60	0.87–7.79	0.088
Carpal tunnel + lumbar spinal stenosis	3.90	1.19–12.80	**0.024**

Statistically significant difference was found between comparison groups with p value less than 0.05.

In the exploratory multivariable Cox model adjusted for age, sex, and hypertension, the combined CTS + LSS category remained independently associated with a higher hazard of scan conversion (adjusted HR 5.95, 95% CI 1.33–26.55; *P* = 0.019), whereas isolated CTS (adjusted HR 1.65, 95% CI 0.35–7.88; *P* = 0.528) and isolated LSS (adjusted HR 1.30, 95% CI 0.22–7.87; *P* = 0.772) were not significant relative to the reference group (neither condition). Hypertension remained independently associated with a lower hazard of conversion (adjusted HR 0.21, 95% CI 0.06–0.75; *P* = 0.016). Age and sex were not statistically significant. (*Table 4*; *Figure 4*, Panel B).

**Table 4:** Multivariable cox regression for imaging conversion

Variable	Adjusted HR	95% CI	*P* value
Age (per year)	1.01	0.94–1.08	0.781
Male sex	1.08	0.28–4.13	0.913
Hypertension	0.21	0.06–0.75	**0.016**
Carpal tunnel only	1.65	0.35–7.88	0.528
Lumbar spinal stenosis only	1.30	0.22–7.87	0.772
Carpal tunnel + lumbar spinal stenosis	5.95	1.33–26.55	**0.019**

Statistically significant difference was found between comparison groups with p value less than 0.05.

## Discussion

The principal finding of this study is that 13.2% of individuals at high risk for cardiac amyloidosis (CA) demonstrated imaging conversion—defined as the emergence of detectable myocardial tracer uptake—from a negative baseline scan within a mean follow-up interval of approximately 3 years. This finding does not necessarily indicate a formal transition to overt ATTR-CA, as lower-grade uptake in particular warrants careful clinical correlation before a definitive diagnosis is established. However, these findings may still be meaningful, as they can identify patients at a stage where treatment may help prevent disease progression. Conversion occurred despite no significant differences in traditional clinical characteristics, cardiac biomarkers, or standard echocardiographic parameters, and was observed across a spectrum of amyloid deposition patterns ranging from localized to diffuse. Given the modest number of conversion events and the exploratory nature of these analyses, findings should be considered hypothesis-generating and interpreted with caution. Nonetheless, these results support the continued use of current guideline-recommended surveillance intervals of 3–5 years, while raising consideration for earlier imaging in individuals who present with both LSS and CTS, a phenotype associated with increased amyloid burden.

Recent epidemiologic data reinforce these findings. De Michieli and colleagues demonstrated that individuals with combined LSS and CTS have a substantially higher 10-year incidence of amyloidosis than those with either condition alone.^6^ Early detection is clinically important given that disease-modifying therapies such as tafamidis, patisiran, and vutrisiran are most effective when initiated before advanced myocardial infiltration.^17–20^ Because extracardiac manifestations—including CTS and LSS— often precede ventricular involvement by 5 to 10 years, extended longitudinal surveillance may be warranted.^6,8^ In the present cohort, only six participants completed a full sequence of three SPECT/CT scans, limiting deeper longitudinal assessment; among them, one patient exhibited conversion 5.7 years after the initial scan in the context of ATTR amyloid originally identified in LAA tissue.

Converters were less frequently hypertensive, aligning with previous work suggesting that autonomic dysfunction, reduced stroke volume, and lower systemic vascular resistance contribute to the characteristic lower blood pressure observed in CA.^21^ Although biomarkers such as NT-proBNP and troponin correlate with disease severity in established ATTR-CA, they may remain stable in early disease.^15^ In this cohort, however, converters more frequently demonstrated a ≥30% rise in NT-proBNP, supporting the potential utility of serial biomarker assessment when interpreted within clinical context.^15^ Nonetheless, given the limited specificity of NT-proBNP and attenuation of its association in multivariable models, biomarker changes alone should not guide surveillance intervals.

Other routinely collected variables—including diabetes, chronic kidney disease, low-voltage ECG, and left ventricular ejection fraction—were not predictive of conversion, consistent with prior literature showing that wall thickness, cavity dimensions, and systolic function often remain normal until later stages of ATTR cardiomyopathy.^22–24^ LAA amyloid deposition emerged as a potentially high-risk feature, as both individuals with confirmed LAA involvement ultimately developed CA, reflecting increasing recognition of atrial amyloid as an early and prognostically adverse phenotype.^25,26^ Tenosynovial amyloid associated with CTS represented the most common path to conversion, with nearly 16% of such patients progressing over the study interval. The combined presence of CTS and LSS independently predicted conversion, whereas either condition alone did not, suggesting that coexisting musculoskeletal manifestations may serve as markers of greater systemic amyloid burden and a transitional stage preceding cardiac involvement. This phenotype may therefore help refine risk stratification and guide more individualized surveillance strategies. Despite these constraints, the clinical relevance of early detection is clear. More than half of converters initiated tafamidis therapy, consistent with evidence that earlier treatment improves morbidity and mortality.^14,19^ Additionally, several genetically tested individuals carried pathogenic TTR variants, underscoring the importance of genotype-informed surveillance.^12^

Although bone scintigraphy remains the gold standard for non-invasive evaluation of CA, it is limited in detecting early or low-burden disease and may yield artifactual uptake in calcified structures.^4^ Emerging imaging modalities—including^124^I-evuzamitide PET/MRI and next-generation SPECT tracers such as ^99^ᵐTc-p5 + 14—offer improved sensitivity, subtype specificity, and whole-body amyloid assessment.^27–29^ These innovations have the potential to enhance diagnostic accuracy, enable earlier detection, and support more personalized management strategies for individuals at risk for cardiac amyloidosis.

## Limitations

This study has several limitations. Its retrospective, single-centre design limits generalizability, and the small number of conversion events raises the possibility of overfitting, making effect estimates less precise. Follow-up scan intervals were not standardized and reflected individualized clinical decision-making, which may have influenced estimates of conversion timing. In addition, clinical outcomes such as mortality, arrhythmias, and heart-failure hospitalizations were not adjudicated, leaving the long-term significance of imaging conversion uncertain.

These findings should be interpreted with caution due to the modest number of conversion events and the potential for overfitting despite conservative covariate selection. Wide confidence intervals for some predictors highlight these limitations. As such, these results should be considered exploratory and hypothesis-generating, warranting validation in larger prospective cohorts. Importantly, the clinical implication is not that all at-risk individuals require uniform serial imaging, but rather that surveillance intensity should be individualized, with closer monitoring prioritized for those presenting with combined CTS and LSS, a phenotype that may signal greater systemic amyloid burden and imminent cardiac involvement.

Despite these limitations, the study is strengthened by the use of only SPECT/CT, which reduces diagnostic ambiguity, and by a meaningful number of biopsy-confirmed amyloid cases that reinforce the accuracy of phenotyping. Larger studies with standardized surveillance protocols and higher event rates are needed to confirm these associations and refine risk-stratification strategies.

## Conclusions

The findings of this study support a risk-adapted rather than uniform surveillance approach for individuals at elevated risk of transthyretin cardiac amyloidosis. As an alternative to applying serial Tc-99m PYP/HMDP SPECT/CT indiscriminately to all at-risk individuals, surveillance intensity could be guided by clinical phenotype—with closer monitoring prioritized for patients exhibiting both CTS and LSS, a combination independently associated with conversion. Notably, clinical markers, biomarkers, and echocardiographic parameters were often unchanged at the time of imaging conversion, underscoring the limitations of relying solely on routine clinical follow-up and reinforcing the added value of periodic imaging in appropriately selected individuals. Prospective studies with standardized surveillance intervals are needed to refine monitoring strategies and determine whether incorporating biomarker trajectories or additional extracardiac features improves risk stratification. Emerging imaging technologies, including next-generation SPECT systems and novel radiotracers, may also influence future surveillance practices, although their clinical impact remains to be defined.

## Clinical perspective

Among individuals at elevated risk for transthyretin cardiac amyloidosis (ATTR-CA), serial Tc-99m PYP/HMDP SPECT/CT identifies a meaningful subset (13.2%) who convert from a negative to a positive scan over approximately 3 years, often in the absence of significant changes in echocardiographic parameters or conventional cardiac biomarkers. The co-occurrence of carpal tunnel syndrome and lumbar spinal stenosis defines a musculoskeletal phenotype associated with substantially increased risk of conversion, suggesting that combined extracardiac manifestations may signal evolving systemic amyloid burden. These findings support individualized surveillance strategies, with particular attention to patients exhibiting both conditions.

## Supplementary Material

qyag089_Supplementary_Data

## Data Availability

The data underlying this article are not publicly available due to institutional privacy regulations and the retrospective nature of the study. De-identified data may be made available upon reasonable request to the corresponding author, subject to institutional review board approval.

## Lead author biography

Saberio Lo Presti, MD, is a staff non-invasive cardiologist in the Tomsich Family Department of Cardiovascular Medicine at the Cleveland Clinic's Miller Family Heart, Vascular & Thoracic Institute, where he joined the medical staff in 2022. His clinical and research interests encompass multimodality cardiac imaging, including Cardiovascular CT, cardiac MRI, echocardiography, and nuclear imaging.

Dr. Lo Presti graduated summa cum laude and first in his class from Universidad de Los Andes School of Medicine, Venezuela. He completed his Internal Medicine residency at Mount Sinai Medical Center, Miami Beach, where he served as Chief Resident, followed by Cardiology Fellowship training at Columbia University Division of Cardiology at Mount Sinai Medical Center and an Advanced Cardiac Imaging Fellowship at the Cleveland Clinic, Ohio, where he served as Chief Fellow. He has particular expertise in cardiac amyloidosis imaging, valvular heart disease, pericardial syndromes, and coronary artery disease characterization.
