# Supplementary material for: Yield and predictors of conversion on serial amyloid nuclear SPECT/CT in at-risk populations for transthyretin cardiac amyloidosis
Source: Eur Heart J Imaging Methods Pract. 2026 May 16;4(1):qyag089. doi: 10.1093/ehjimp/qyag089 (PMC13247585; doi:10.1093/ehjimp/qyag089)
Supplement: qyag089_Supplementary_Data [file qyag089_supplementary_data.docx]

**Supplemental Table 1: Number and Percentage of Imaging conversions per year.**

| Year | Number of conversions (%) |
| --- | --- |
| 2 | 2 (14.3%) |
| 3 | 6 (42.8%) |
| 4 | 3 (21.4%) |
| 5 | 2 (14.3%) |
| >5 | 1 (7.15%) |

**Supplemental Table 2. Biopsy site distribution by conversion status (n=47).**

| Biopsy Site | Total (n=47) | No Conversion (n=39) | Conversion (n=8) |
| --- | --- | --- | --- |
| Tenosynovial tissue | 32 (68.1%) | 27 (69.2%) | 5 (62.5%) |
| Ligamentum flavum | 8 (17.0%) | 7 (18.0%) | 1 (12.5%) |
| Left atrial appendage (LAA) | 3 (6.4%) | 1 (2.6%) | 2 (25.0%) |
| Other sites | 4 (8.5%) | 4 (10.2%) | 0 (0%) |

**Supplemetal Table 3. Genetic and Disease-Modifying Therapy Characteristics Among Patients with Conversion (n = 14).**

| **Genetic Characteristic** | **n (%)** |
| --- | --- |
| **TTR mutation present** | 4 (28.6%) |
| ├─ Val142Ile | 2 (14.3%) |
| └─ Other TTR variants | 2 (14.3%) |
| **Wild-type (no mutation identified)** | 10 (71.4%) |
| **Therapy** | **n (%)** |
| **Tafamidis** | 9 (64.3%) |
| **Diflunisal** | 1 (7.1%) |
| **Vutrisiran** | 3 (21.4%) |
| **No disease-modifying therapy** | 1 (7.1%) |

**Supplemental Table 4. Conversion Rates by At-Risk Subgroup.**

| **At-Risk Subgroup** | **Total** | **Converters** | **Conversion Rate** |
| --- | --- | --- | --- |
| Extracardiac biopsy-proven amyloid | 47 | 8 | 17.0% |
| TTR mutation carriers | 19 | 4 | 21.1% |
| High clinical suspicion | 42 | 2 | 4.8% |

*Subgroups are not mutually exclusive; some patients met more than one at-risk criterion. Percentages reflect conversions within each subgroup independently.*
